# Principles of empiric antimicrobial usage and dosing: Lessons learned

**DOI:** 10.1002/ccr3.5594

**Published:** 2022-03-17

**Authors:** Kathryn M. Lalonde, Cameron Black, John C. Lam

**Affiliations:** ^1^ Department of Medicine University of Calgary Calgary Alberta Canada; ^2^ Faculty of Pharmacy & Pharmaceutical Sciences University of Alberta Edmonton Alberta Canada

**Keywords:** antibiotics, clinical reasoning, infectious diseases

## Abstract

A lack of clinical response to empiric antimicrobials behooves the clinician to reflect further on diagnostic considerations. When prescribing antibiotics, determining the correct dose, most optimal route of administration, and considering the pharmacokinetic properties of the drug with respect to clinical and patient factors are crucial.

## Abstract 2

Improving antibiotic usage is paramount. Antibiotic prescribing is a purposeful process, requiring a clinical indication, determination of the optimal drug dosage/route of administration, and consideration of clinical pharmacokinetics in a patient.

## 1 INTRODUCTION

The advent of and access to antimicrobials have been critical in the treatment of infectious diseases over the last century. Increasing access to antibiotics has contributed to inappropriate usage and antimicrobial resistance. Optimal prescribing of antibiotics consists of selecting an appropriate antimicrobial based on a pertinent clinical indication and administered via the correct route, dose, frequency, and duration. Herein, we present a patient who was trialed with multiple courses of antibiotics without diagnostic clarity or clinical improvement. Retrospectively, the dosing and route of medication administration were not optimized, which may have contributed to a protracted course of disease.

## 2 CASE PRESENTATION

A 29‐year‐old multiparous female with diabetes presented with a 3‐month history of a painful, swollen, right‐sided erythematous breast lump, which progressed after her child ran into her chest. Owing to increased tenderness, induration, and drainage from her breast, she sought medical attention. Through daily visits to the emergency room, the patient received 5 days of intravenous cefazolin 2 g once daily with 1 g oral probenecid. When there was no clinical improvement, her antibiotics were changed to an 8‐day course of clindamycin 600 mg intravenously daily. As the change of antibacterials did not reduce the tenderness and induration, she underwent a breast biopsy to assess for malignancy. Pathology demonstrated fat necrosis with non‐caseating granulomatous changes in the breast parenchyma and Gram‐positive bacilli within the rounded cystic spaces. There were no features of malignancy, and a diagnosis of cystic neutrophilic granulomatous mastitis was made, prompting a cessation of all antibacterials.

Unfortunately, drainage of her breast continued after discontinuation of antibiotics and she was commenced on a 10‐day course of doxycycline 100 mg orally twice daily, followed by a 4‐day course of trimethoprim/sulfamethoxazole 160/800 mg orally twice daily. When the usage of trimethoprim/sulfamethoxazole did not yield clinical improvement, an infectious diseases consultation was arranged.

On histology, the presence of Gram‐positive bacilli and lobulo‐centric inflammatory infiltration was suspicious for *Corynebacterium* species as a contributor to the pathological diagnosis of granulomatous mastitis. The identification of lipophilic *Corynebacterium* species is challenging and requires special media and, at times, genomic sequencing for confirmation. Acknowledging the association between *Corynebacterium* species and granulomatous mastitis, this patient was initiated on a trial of doxycycline 100 mg orally twice daily and clindamycin 450 mg orally thrice daily to facilitate penetration of lipophilic antibacterials into breast tissue. Her case was also discussed with a breast surgeon, given the paucity of literature guiding the treatment of granulomatous mastitis.^1^ She will be followed up routinely by both a breast surgeon and an infectious diseases consultant to assess for tolerance of therapy and clinical improvement.

## 3 DISCUSSION

While a lack of clinical response to empiric therapies is frustrating for both patient and provider, recognition of the same is a critical skill set of a diagnostician. Medical education emphasizes the need for constantly re‐evaluating a differential diagnosis as more data are gathered. A trial of empiric therapy, successful or not, represents important information in achieving diagnostic clarity. The case described herein illustrates key concepts to question when empiric antibacterials are not effective.

Firstly, the breast biopsy obtained was imperative for assessing the possibility of malignancy. When tissue pathology revealed granulomatous tissue and Gram‐positive bacilli, the multiple short courses of antibacterials that the patient was placed on did not result in clinical improvement. Although she did not have serious adverse effects of antibacterial therapy, retrospective reflection on her unusual clinical course suggests earlier consideration of a new treatment plan, potentially through multidisciplinary consultation, may have reduced exposure to additional courses of potentially harmful antibacterials.^2^

Another consideration with antibiotic use is an understanding of oral bioavailability. Blood and target tissue concentrations are equivalent when using a highly bioavailable oral antibiotic agent compared with its intravenous counterpart.^3^ Multiple antimicrobials including clindamycin, metronidazole, linezolid, fluoroquinolones, tetracyclines, rifampin, trimethoprim/sulfamethoxazole, and triazole antifungals have an oral bioavailability in excess of 90%.^1^ The benefits of employing a highly bioavailable oral agent over its intravenous counterpart include decreased time needed for preparation and administration, reduction in intravenous line‐associated complications (including infections and thrombophlebitis), decreased cost of health professionals needed to administer the drug, and early discharge from a treatment facility.^4^

By utilizing intravenous clindamycin, our patient was required to attend the hospital daily, a relevant impediment to her ability to attend work and generate income, particularly when an oral equivalent was available. Importantly, her intravenous clindamycin was not dosed thrice daily owing to the inability to facilitate multiple visits per day for parenteral antimicrobial therapy, therefore rendering it potentially ineffective. In patients with a functional gastrointestinal tract, the use of a highly bioavailable oral agent is almost always preferred over the use of its intravenous equivalent.

Lastly, effective antibiotic therapy requires consideration of pharmacokinetic properties, as drug concentrations must be adequate within the infected tissue for successful treatment of infection. For example, treatment of central nervous system infections requires antimicrobials that can successfully cross the blood–brain barrier. In our patient, clindamycin and doxycycline were utilized owing to their lipophilic properties and enhanced ability to penetrate breast tissue.^5^

## 4 CONCLUSION

Antibiotics are essential drugs for bacterial infections. Similar to other medications, a lack of clinical response to empiric antimicrobial usage behooves the clinician to reflect further on diagnostic considerations. During challenging cases of diagnostic uncertainty, multidisciplinary consultations may be helpful in formulating a revised treatment plan. When prescribing antibiotics, it is crucial to review the correct dose, determine the most optimal route of administration, and consider the pharmacokinetic properties of the drug with respect to individual clinical and patient factors.

## CONFLICT OF INTEREST

None.

## AUTHOR CONTRIBUTIONS

KML and CB wrote the initial draft of the manuscript and all authors contributed to its revision. JCL oversaw manuscript preparation and revisions. All authors read and approved the manuscript.

## CONSENT

The patient described provided written informed consent for this case to be prepared and published.

## Data Availability

Data sharing not applicable – no new data generated.
